# The gut microbiome as a potential predictive biomarker for breast cancer: emerging association and geographic differences

**DOI:** 10.3389/fonc.2025.1666830

**Published:** 2025-12-12

**Authors:** Byeongsang Oh, Gillian Lamoury, Susan Carroll, Marita Morgia, Frances Boyle, Nick Pavlakis, Stephen Clarke, Alexander Guminski, Alexander Menzies, Connie Diakos, Katrina Moore, Sally Baron-Hay, Thomas Eade, Mark Molloy, Michael Back

**Affiliations:** 1 Northern Sydney Cancer Center, Royal North Shore Hospital, St Leonards, NSW, Australia; 2 The Mater Hospital, North Sydney, NSW, Australia; 3 Faculty of Medicine and Health, University of Sydney, Sydney, NSW, Australia; 4 Genesis Care, North Shore Health Hub, St Leonard, NSW, Australia; 5 Bowel Cancer and Biomarker Laboratory, Kolling Institute, St Leonard, NSW, Australia

**Keywords:** breast cancer, gut microbiome, predictive biomarker, geographical variation, estrogen metabolism, precision oncology

## Abstract

**Background:**

The gut microbiome may influence breast cancer (BC) development by modulating estrogen metabolism, immune responses, and microbial metabolites. Altered microbial patterns have been reported in BC, but their value as predictive biomarkers remains uncertain.

**Methods:**

We reviewed 13 case–control studies that compared gut microbiome composition in women with and without BC, focusing on diversity, compositional shifts, and geographic variation.

**Results:**

Reduced microbial richness (*alpha diversity*, the number and balance of bacterial species) was observed in more than half of the studies, although findings were not uniform. Differences in community composition (*beta diversity*) were common. Across studies, BC was consistently associated with elevated *Bacteroides* and reduced *Faecalibacterium*, a genus linked to anti-inflammatory effects. Other recurrent findings included enrichment of *Eggerthella* and *Blautia* in BC, though results for several taxa were inconsistent. Geographic variation was evident: *Eggerthella* was enriched in U.S. cohorts, *Blautia* in European cohorts, and in Chinese cohorts, *Prevotella* was elevated while *Akkermansia* was reduced.

**Conclusions:**

Despite heterogeneity, converging evidence supports reduced diversity and shifts in select taxa, particularly enrichment of *Bacteroides* and depletion of *Faecalibacterium*, as emerging features of the BC microbiome. Geographic differences underscore the influence of host and environmental factors. These findings suggest biomarker potential but highlight the need for larger, longitudinal, and standardized studies to establish causality and clinical utility.

## 1 Introduction

Breast cancer (BC) is the most prevalent cancer among women globally and remains a leading cause of cancer-related morbidity and mortality, despite significant advancements in early detection and treatment (1). Innovations such as advanced imaging techniques, minimally invasive surgeries, targeted therapies, immunotherapies, personalized medicine, radiation therapy, and multidisciplinary approaches have contributed to improved survival outcomes (2). However, approximately 20-30% of women diagnosed with early-stage BC, depending on the subtype, experience recurrence, often manifesting as metastatic disease (3, 4). This highlights the critical need for enhanced early detection strategies and innovative prognostic tools to improve therapeutic efficacy and survival outcomes.

Emerging evidence highlights the pivotal role of the gut microbiome in several cancers, including BC, where dysbiosis has been implicated in disease initiation, progression, and therapeutic response (5–9). Current evidence suggests that the gut microbiome influences BC risk by modulating systemic estrogen levels (10), metabolite production (11), and inflammatory responses. Elevated estrogen levels are well-documented risk factors for BC, with β-glucuronidase enzymes produced by certain gut bacteria facilitating estrogen reabsorption into the bloodstream, thereby contributing to BC pathogenesis (10, 12). Recent mechanistic studies also demonstrate that gut microbiota can regulate steroid hormone activity, immune modulation, and therapeutic response in BC, further underscoring their potential role as biomarkers and therapeutic targets (7, 13–15).

Several reviews have investigated the relationship between gut microbiome composition and BC. A systematic review of 10 case studies reported that BC patients exhibit decreased relative abundance of beneficial bacteria such as *Prevotellaceae, Ruminococcus, Roseburia* inulinivorans, and *Faecalibacterium prausnitzii*, alongside increased abundance of *Bacteroides* and *Erysipelotrichaceae* (16). However, findings across studies remain inconsistent, with variability linked to cancer stage, molecular subtype (11, 17, 18), menopausal status (10, 19), ethnicity, body mass index (20), diet (13), and medication use (21). Moreover, although prior reviews have highlighted mechanistic links between microbiota, estrogen metabolism, and BC progression, they have not adequately addressed how these associations may differ across geographical regions. This review, therefore, aims to fill this gap by synthesizing evidence on gut microbiome variations in BC across China, the USA, and Europe. By critically examining geographical differences, we seek to identify consistent microbial signatures, evaluate their potential as predictive biomarkers (22), and highlight directions for future research. Ultimately, this analysis may contribute to the development of microbiome-informed strategies that support personalized approaches to BC management.

## 2 Methods

We performed a mini-review of the literature through structured searches of PubMed, Medline, and ScienceDirect from database inception through December 2024. The search strategy included the terms “gut microbiome,” “gut microbiota,” “breast cancer,” and related synonyms. Reference lists of eligible articles were also screened. Eligible studies were peer-reviewed, English-language, human case–control studies comparing gut microbiome composition in adult women with BC and healthy controls. We excluded animal and preclinical studies, reviews, editorials, conference abstracts, and non-English publications. Data extracted from each study included participant characteristics, geographic setting, sequencing platform, measures of microbial diversity, and taxonomic findings. Owing to heterogeneity in study design, sequencing methodology (16S rRNA *vs*. metagenomics), and reported outcomes, quantitative meta-analysis was not feasible. Instead, we undertook a narrative synthesis, with emphasis on consistent and divergent findings and on geographic patterns across cohorts from China, Europe, and the United States.

## 3 Results

### 3.1 Demographics and characteristics of studies

The studies were published from 2018 to 2024 and involved research from various regions, including China (n=5) (19, 23–26), the USA (n=3) (27–29), the Netherlands (n=1) (30), France (n=1) (31), Poland (n=1) (32), Taiwan (n=1) (33), and Ghana (n=1) (34). The study population includes premenopausal (n=2) (23), postmenopausal (n=1) (19), and mixed pre/post-menopausal (n=7) BC populations (33). Three studies did not describe the menopausal status (24, 25, 34). Specific populations were BC with/without bone metastasis (n=1) (25) while two studies not reported study populations were either pre- or post-menopausal BC. Sample sizes ranged from 22 to 379. Common participants’ immunohistochemistry was ER+ (n=10) followed by HER2+ (n=5), and triple-negative breast cancer (TNBC) (n=2). Four studies did not report their participants’ immunohistochemistry data (24). Feces were collected and universally used across studies for microbiota analysis, while each study collected saliva (28) and blood (24) in addition to feces. Microbiota analysis was commonly performed with 16S rRNA sequencing (n=11) (23–25, 33, 34) while two were conducted with shotgun metagenomics (19, 29) and one study used PacBio (26) (**Table 1**).

**Table 1:** Characteristics of studies.

Author year country	Study population	Sample size, age		Immuno- histochemistry	Biospecimen collection	Microbiota analysis	Results
		BC	HC				
Aarnoutse et al., 2021 Netherlands (30)	Post-menopausal BC	81 62 yrs	67 62 yrs	ER + Her 2 −	Faeces	16S rRNA region v4	While an increased relative abundance of ***Dialister*** and ***Veillonellaceae*** was observed in BC patients scheduled for adjuvant treatment—potentially attributable to prophylactic antibiotic administration—no significant differences in microbiota richness, diversity, or overall composition were identified between postmenopausal BC patients and control groups.
Bobin-Dubigeon et al., 2021 France (31)	Newly diagnosed Pre and post-menopausal BC	25 63 yrs	30 53 yrs	ER + Her 2 −	Faeces	16S rRNA region v3-v4	Alpha diversity was significantly lower in the BC group compared to controls. A tendency towards a decreased relative abundance **of *Odoribacter* sp.*, Butyricimonas* sp.*, and Coprococcus* sp.** was observed. These findings suggest that BC patients may exhibit distinct differences in their intestinal bacterial composition compared to healthy individuals.
Zeber-Lubecka et al., 2024 Poland (32)	Pre, peri and post-menopausal BC 174	Pre/peri-BC (n=47) Post-BC (n=41)	Pre/peri (n=51) Post (n=35)	ER+(n=54) HER2+(n=21) TNBC (n=12)	Faeces	Shotgun metagenomics	The study results did not identify an association between changes in the overall microbiota composition and selected taxa with menopausal status in BC patients and controls. However, the findings confirmed distinct differences in the gut microbiota between premenopausal and postmenopausal BC patients and their respective control groups
Altinok Dindar et al., 2023 USA (27)	Pre and post-menopausal BC	42 6o yrs	44 58 yrs		Faeces	16S rRNA region v4	Associations between significant microbial genera identified from BC patients and healthy control groups highlight the potential of the gut microbiome as a valuable source of biomarkers for breast cancer risk assessment.
Shrode et al., 2023, USA (29)	Pre and post-menopausal BC	22 67 yrs	19 56 yrs	ER + (n=18) HER2+ (n=1) TNBC (n=4)	Faeces	16S rRNA region v3-v4 Metagenomic sequencing	The study observed gut dysbiosis in BC patients, characterized by the depletion of SCFA-producing gut bacteria, suggesting their potential role in the pathobiology of BC. A deeper mechanistic understanding of gut bacterial dysbiosis in BC could pave the way for refined strategies in prevention and treatment.
McCune et al., 2024 USA (28)	BC and DCIS	BC (n= 66), 56 yrs DCIS (n=29) 54 yrs	42 53 yrs	N/R	Faeces and Saliva	16S rRNA region v4	The study identified several distinguishing features in the gut microbiota associated with BC and DCIS, suggesting that alterations in the gut microbiota may serve as a foundation for interventions targeting the gut microbiome to enhance treatment outcomes and improve long-term prognosis.
Zhu et al., 2018 China (19)	Premenopausal BC	N=62 Pre (n=18) 37 yrs	71 Pre (n=25) 35 yrs	N/R	Faeces	Shotgun metagenomics	The microbiota profiles differ between postmenopausal BC in women and healthy controls but not in premenopausal women. The gut microbiota may regulate or respond to host immunity and metabolic balance.
	Postmenopausal BC	Post (n=44) 57 yrs	Post (n=46) 56 yrs		
He et al., 2021 China (23)	Premenopausal BC	54 39 yrs	28 37 yrs	N/R	Faeces	16S rRNA region v3-v4	The study revealed significant differences in the composition and symbiosis of gut microbiota between premenopausal BC patients and healthy premenopausal women. The abundance of short-chain fatty acid (SCFA)– producing bacteria and key SCFA-producing enzymes were significantly reduced in BC patients. Furthermore, *Pediococcus and Desulfovibrio* were identified as potential microbial markers capable of distinguishing premenopausal breast cancer patients from healthy individuals.
Hou et al., 2021 Taiwan (33)	Pre and post-menopausal BC	200 Pre (n=100) 41 yrs Post (n=100) 60 yrs	67 Pre (n=50) 35 yrs Post (n=17) 61 yrs	ER+ (n=167) HER+ (n=50)	Faeces	16S rRNA region v4	These findings provide the first evidence that the gut microbiota in premenopausal BC patients differs significantly from that in postmenopausal patients, highlighting menopausal-specific microbial markers for diagnosis and investigation. This research underscores the potential for a non-invasive approach to BC detection and offers a novel strategy for preventing premenopausal BC.
Ma et al., 2022 China (24)	BC	BC (n=26) 49 yrs	HC (n=20) 46 yrs	N/R	Faeces	16S rRNA region v3-v4	This study reveals a decreasing trend in gut microbiota diversity of BC and benign breast legion subjects compared to healthy individuals. Compared with healthy individuals, the levels of *Porphyromonas and Peptoniphilus* were higher in BC patients, whereas *Escherichia and Lactobacillus* were more enriched in the benign breast lesion (BBL) group. This indicates that patients with BC and BBLs may undergo significant changes in intestinal microbiota.
Wenhui et al., 2022 China (25)	BC with no bone metastasis (BN)	BC (n=32) 52 yrs	25 54 yrs	ER+ (n=23) HER+ (n=24)	Faeces	16S rRNA region v4	This study demonstrated that variations in gut microbiota are associated with BC occurrence and bone metastasis, providing attractive targets for developing therapeutic and diagnostic methods. Streptococcus, *Campylobacter and Moraxellaceae* showed higher abundances in BNs and BMs than in HCs. The lack of *Megamonas and Akkermansia* in the BM compared with those in the NC and BN groups was considered related to bone metastasis.
	BC with bone metastasis (BM)	BM(n=22) 50 yrs		ER+ (n=16) ER+ (n=14)	Faeces
Jiang et al, 2023 China (26)	Menopausal Pre-menopause	43	30	ER+ (n=34) HER2+ (n=21)	Faeces	PacBio -16S full length	This study suggests that BC is associated with changes in the composition and function of intestinal flora. These microflora and functional differences may be biomarkers or new targets for diagnosing and treating BC.
Byrd et al., 2021 Ghana (34)	Pre and post-menopausal BC Antibiotic use	379 50 yrs (18–74 yrs)	414 46 yrs	ER + Her 2 −	Faeces	16S rRNA region v4	Fecal bacteria characteristics are associated with BC and non-malignant breast disease. *Bacteroides and Flavonifractor* are positively associated with BC. *Romboutsia and Coprococcus, Prevotella,Faecalibacterium, Eubacterium, Dorea, and Collinsella* are inversely associated with BC.

BC, Breast cancer; DCIS; Ductal carcinoma *in-situ*; HC, Healthy control; F, Faeces; **↓** decreased/low, **↑**increased/high, NS, No significant difference; HR, hormone receptor; HER2, human epidermal growth factor receptor 2; TNBC, triple negative breast cancer; Firmicutes/Bacteroidetes, LEfSe, Linear discriminant analysis effect size; AUC, Area under receiver operating curve; NR, Not reported; DCIS, Ductal carcinoma *in situ*; N/R, Not reported

### 3.2 Gut microbiota diversity and composition between the BC and HC

#### 3.2.1 Diversity

Of 13 studies, 7 reported lower alpha diversity in BC compared with HC, while 2 reported higher diversity and 4 reported no significant differences. Variability was partly attributed to menopausal status and diversity indices applied (Shannon v.s. Chao). For beta diversity, 7 studies found significant differences in overall microbial structure between BC and HC, whereas 4 reported no differences (**Table 2**).

**Table 2:** Gut microbiome differences between BC and controls.

	Diversity		Pylum		Genus/Genera		Species	
	Alpha	Beta	BCa	Control	BCa	Control	BCa	Control
Aarnoutse et al., 2021 Netherlands (30)	NS	NS	NS		NS	NS		
Bobin-Dubigeon et al., 2021 France (31)	**↓**	NR	**↑** Firmicutes	**↑**Bacteroidetes	**↑***Clostridium* cluster IV **↑***Clostridium* cluster XIVa **↑***Blautia* sp.	**↑***Bifidobacterium* sp., **↑***Odoribacter* sp., **↑***Butyricimonas*.sp., **↑***Coprococcus* sp.		
Zeber-Lubecka et al., 2024 Poland (32)	NS -Pre	NS	**NS-**F/B ratio **↓***Actinobacteria*	**↑** *Synergistetes*	**↑** *Coprobacter* **↓** *Gemmiger* **↓** *Bifidobacterium* **↓** *Ruthenibacterium* **↓** *Anaeromassilibacillus* **↓** *Anaeromassilibacillus*		**↓** *Collinsella_massiliensis* **↓** *Gemmiger_formicilis* **↓** *Collinsella_stercoris*	
	NS -Post	NS			**↑***Coprobacter* **↑***Parabacteroides* **↑***Dorea* **↑***Blautia* **↑***Barnesiella* **↑***Bacteroides* **↓***Agathobaculum* **↓***Harryflintia* **↓***Enterorhabdus* **↓***Rothia* **↓***Allisonella* ↓*Bifidobacterium*		**↑** *Coprobacter_fastidiosus* **↑** *Bacteroides_thetaiotaomicron* **↑** *Parabacteroides_distasonis* **↑** *Blautia_obeum* **↑** *Phascolarctobacterium_faecium* **↑** *Clostridium_sp_CAG_167* **↑** *Barnesiella_intestinihominis* **↑** *Dorea_formicigenerans* **↓** *Collinsella_intestinalis* **↓** *Agathobaculum_butyriciproducens*	
	Pre and post							
Altinok Dindar et al., 2023 USA (27)	**↓**	NS			**↑***Acidaminococcus*, **↑***Tyzzerella*, **↑***Hungatella*	**↑***Christensenellaceae*, **↑***UCG-005*, **↑***Oscillospirales*, **↑***NK4A14 group*, **↑***Dialister*, **↑***Gastranaerophilales*, **↑***Romboutsia*, **↑***Coriobacteriales*, **↑***Anaerofilum*, **↑***Flavobacterials*		
Shrode et al., 2023, USA (29)	NS	Different	**↑** Firmicutes **↑**F/B ratio	**↑**Bacteroidetes	**↑** *Intestinibacter* **↑** *Faecalitalea* **↑** *Eggerthella* **↑** *Turicibacter*	**↑** *Erysipelotrichaceae UCG 003* **↑** *Lachnospiraceae NK$A136_group*	**↑***Intestinibacter bartlettii* **↑***Faecalitalea* sp*ecies* **↑***Eggerthella lenta* Random forest analysis **↑***Oscillospiraceae* sp*ecies* **↑***Actinomyces* sp*ecies* **↑***Eggerthella lenta* **↑***Faecalitalea* sp*ecies* **↑***Intestinibacter bartlettii* **↑***Blautia* sp*ecies*	**↑***Parabacteroides merdae* **↑***Erysipelotrichaceae UCG 003 bacterium* **↑***Faecalibacterium prausnitzii*, **↑***Erysipelotrichaceae UCG 003 bacterium*, **↑***Lachnoclostridium edouardi*, **↑***Oscillibacter* sp*ecies*, **↑***Lachnospiraceae UCG 010* sp*ecies*, **↑***Lachnospira pectinoshiza*, **↑***Alistipes* sp*ecies*, **↑***Parabacteroides merdae*
McCune et al., 2024 USA (28)	BC **↓**	Different	**↑***Tenericutes* **↓**F/B ratio	**↑** *Firmicutes*	**↑***Bacteroides*, **↑***Finegoldia*	**↑** *Anaerostipes* **↑** *Coprococcus*		
					**↑***Coprobacillus*, **↑***Parabacteroides*, **↑***Streptococcus*, **↑***WAL*, **↑***Corynebacterium*, **↑***Anaerococcus*, **↑***Acidaminococcus*, **↑***Eggerthella*, **↑***Peptoniphilus* **↑***Bacteroides*, **↑***Finegoldia*			
	DCIS NS	Different	**↑***Actinobacteria* **↓**F/B ratio		**↑***Megamonas*, **↑***Corynebacterium*, **↑***Varibaculum;* **↑***Dialister*	**↑** *Faecalibacterium*		
					**↑***Megamonas*, **↑***Finegoldia*, **↑***Varibaculum;* **↑***Peptoniphilus*, **↑***Corynebacterium*, **↑***Anaerococcus*, **↑***Porphyromonas* **↑***Actinomyces*, **↑***Odoribacter.*, **↑***WAL*, **↑***Streptococcus* **↑***Eggerthella*	**↑** *Prevotella*		
Zhu et al., 2018 China (19)	Pre Chao-NS Shannon-**↑**	Pre NS					NS	NS
	Post Chao-↑ Shannon-NS	Post **↑**					14 optimal species markers- **Post-menopause** **↑***Fusobacterium_varium* **↑***Shigella_sp_D9* **↑***Desulfovibrio_piger* **↑***Escherichia_sp_1_1_43* **↑***Shigella_sonnei* **↑***Eubacterium_eligens* **↑***Escherichia_sp_3_2_53FAA* **↑***Vibrio_cholerae* **↑***Acinetobacter_baumannii* **↑***Proteus_mirabilis* **↑***Fusobacterium_nucleatum* **↑***Campylobacter_concisus* **↑***Escherichia_coli* **↑***Porphyromonas_uenonis* Post menopausal 38 species were enriched in patients, including **↑***Escherichia_coli* **↑***Shigella_sp_D9* **↑***Escherichia_sp_3_2_53FAA* **↑***Shigella_sonnei* **↑***Escherichia_sp_1_1_43* **↑***Proteus_mirabilis* **↑***Shigella_boydii* **↑***Vibrio_cholerae* **↑***Escherichia_fergusonii* **↑***Escherichia_sp_4_1_40B* **↑***Shigella_flexneri* **↑***Acinetobacter_baumannii* **↑***Escherichia_sp_TW09276* **↑***Actinomyces_sp_HPA0247* **↑***Acinetobacter_johnsonii* **↑***Providencia_rettgeri* **↑***Lactobacillus_mucosae* *unclassified_Citrobacter_sp._30_2* **↑***Citrobacter_sp_30_2* **↑***Porphyromonas_uenonis* **↑***Citrobacter_koseri* **↑***Desulfovibrio_piger* **↑***Klebsiella_sp_1_1_55* **↑***Enterococcus_gallinarum* **↑***Salmonella_enterica* **↑***Erwinia_amylovora* **↑***Sodalis_glossinidius* **↑***Acinetobacter_radioresistens* **↑***Fusobacterium_varium* **↑***Acidaminococcus_intestini* **↑***Prevotella_amnii* **↑***Yersinia_enterocolitica* *unclassified_* **↑***Fusobacterium* *unclassified_Prevotella_sp._oral_taxon_299* **↑***Anaerococcus_vaginalis* **↑***Shewanella_putrefaciens* **↑***Fusobacterium_nucleatum* **↑***Escherichia_sp_TW11588* **7 species were reduced in patients, including** **↓***Eubacterium eligens* **↓***Escherichia_albertii* **↓***Campylobacter_concisus* **↓***Roseburia inulinivorans* **↓***Brucella_melitensis* **↓***Lactobacillus vaginalis* **↓***unclassified_Enterobacteriaceae_bacterium_9_2_54FAA*	**Tenfold cross-validation** **↑** *Eubacterium eligens* **↑** *Roseburia inulinivorans*
He et al., 2021 China (23)	NS	Different	**↑**F/B ratio		**↑** *Providencia* **↑** *Romboutsia* **↑** *Desulfovibrio*	**↑** *Pediococcu* ***s*** **↑** *Enterococcus* **↑** *Fusobacterium* **↑** *Megamonas* **↑** *Collinsella* **↑** *Abiotrophia* **↑** *Allisonella*	**↑** *Providencia_vermicola* **↑** *Dialister_invisus* **↑** *Romboutsia_sedimentorum*	**↑** *Megamonas_funiformis* **↑** *Bacteroides_plebeius* **↑** *Dialister_succinatiphilus*
Hou et al., 2021 Taiwan (33)	Pre **↓**	NS		*Actinobacteria*	**↑** *Haemophilus* **↑** *Fusobacterium* **↑** *Sutterella* **↑** *Bacteroides_* **↑** *Ruminococcus* **↑** *Prevotella_*	**↑***Dialister* **↑***Streptococcus* **↑***Megasphaera* **↑***Coprococuccus* **↑***Parabacteroides* **↑***Collinsella* **↑***Bifidobacterium* **↑***Akkermansia* **↑***Alistipes*, **↑***Enterococcus* **↑***Rothia*, **↑***Oxalobacter*, **↑***Enterobacter*	**↓***Bifidobacterium longum*, **↓***Bifidobacterium bifidum*, **↓***Bifidobacterium adolescentis* **↑***Anaerostipes* **↑***Bacteroides fragilis*	**↑***Collinsella aerofaciens* **↑***Bifidobacterium longum*, **↑***Eubacterium bifome* **↑***Bifidobacterium adolescentis* **↑***Akkermansia muciniphila* **↑***Parabacteroides distasonis* **↑***Bifidobacterium bifidum*, **↑***Alistipes indistinctus* **↑***Rothia mucilaginosa*
	Post NS	Different	*Proteobacteria*	*Verrucomicrobia*	**↑** *Actinomyces* **↑** *Mitsuokella* **↑** *Haemophilus* **↑** *Sutterella* ***↓*** *Phascolarctobacterium*	**↑***Akkermansia* **↑***Phascolarctobacterium* **↑***Streptococcus* **↑***Ruminococcus* **↑***Bilophila* **↑***Alistipes*, **↑***Oxalobacter*, **↑***Eggerthella*	**↑***Mitsuokella multacida* **↑***Haemophilus parainfluebzae* **↑***Bifidobacterium longum*, **↓***Bifidobacterium bifidum*, **↓***Bifidobacterium adolescentis* **↑***Anaerostipes* **↓***Bacteroides fragilis* ***↓****Akkermansia muciniphila*	**↑***Akkermansia muciniphila* **↑***Collinsella aerofaciens* **↑***Bacteroides coprophilus* **↑***Parabacteroides distasonis* **↑***Eubacterium bifome* **↑***Oxalobacter* formigenes **↑***Alistipes indistinctus* **↑***Eggerthella lenta* **↑***Alistipes massiliensis*
	Both Pre+ post				**↑** *Sutterella* **↑** *Haemophilus parainfluenzae*	**↓** *Sutterella* **↓** *Haemophilus parainfluenzae*	**↓** *Faecalibacterium prausnitzii* **↓** *Ruminococcus gnavus* **↓** *Rothia mucilaginosa*	**↑** *Faecalibacterium prausnitzii* **↑** *Ruminococcus gnavus* **↑** *Rothia mucilaginosa*
Ma et al., 2022 China (24)	**↓**	Different	**↓**Firmicutes **↑** Bacteroidetes		**↑** *Prevotella* **↑** *Porphyromonas* **↑** *Peptoniphilus* **↑** *Megamonas*	**↑***Eubacterium* **↑***Alistipes* **↑***Christensenella* **↑***Oxalobacter* **↑***Collinsella* **↑***Acidaminococcus* **↑***Tissierella* **↑***Butyricimonas* **↑***Hydrogenoanaerobacterium* **↑***Cloacibacillus*, **↑***Asaccharobacter*		
Wenhui et al., 2022 China (25)	BC with no metastasis NS	NS	**↓** Bacteroidetes		**↑**Proteobacteria, **↑***Staphylococcus*, **↑***Campylobacter*, **↓***Paraprevotella*	**↑** *Paraprevotella*		
	Bone metastasis **↓**	Different	**↓** Firmicutes		**↑**Bacilli, **↑**Veillonella, **↑***Streptococcus*, **↑***Campylobacter*, **↑**Acinetobacter, **↑**Collinsella	**↑**Megamonas, **↑***Clostridia*, **↑***Akkermansia*, **↑**Gemmiger, **↑**Paraprevotella		
Jiang et al, 2023 China (26)	**↑**	NS	**↑** Firmicutes	**↑**Bacteroidetes	**↑***Lachnospira*, **↑***Ruminococcaceae_UCG_013*, **↑***Family_XIII_UCG_001*, **↑***Coprococcus*, **↑***Ruminococcaceae_UCG_002*, **↑***Christensenella*, **↑***Butyricicoccus*, **↑***Erysipe lotrichaceae_UCG_003*, **↑***Lachnospiraceae_NK4A136_group*, **↑***Christense nellaceae_R_7_group*, **↑***Tyzzerella* **↑***Faecalibacterium*	**↑***Bacteroides*, **↑***Veillonella*, **↑***Clostridium*, **↑***Ruminococcus_torques_group*, **↑***Eggerthella*		
Byrd et al., 2021 Ghana (34)	**↓**BC				**↑***Bacteroides*, **↑***Flavonifractor* **↓***Romboutsia and***↓***Coprococcus,Prevotella*, **↓***Faecalibacterium*, **↓***Eubacterium*, **↓***Dorea*, **↓***Collinsella*			

### 3.3 The difference in gut microbiota composition between the BC and HC

#### 3.3.1 Phylum level

At the phylum level, dysbiosis in BC was variably reported. Four studies (23, 26, 29, 31) observed an increased abundance of Firmicutes and higher Firmicutes/Bacteroidetes (F/B) ratios in BC compared with controls, while three studies (24, 25, 28) reported decreased ratios. The remaining investigations found no significant or inconclusive differences. Actinobacteria findings were also inconsistent, with some studies reporting enrichment (28) and others depletion (32), underscoring methodological heterogeneity and population-specific effects.

#### 3.3.2 Genus level

**Figure 1:**
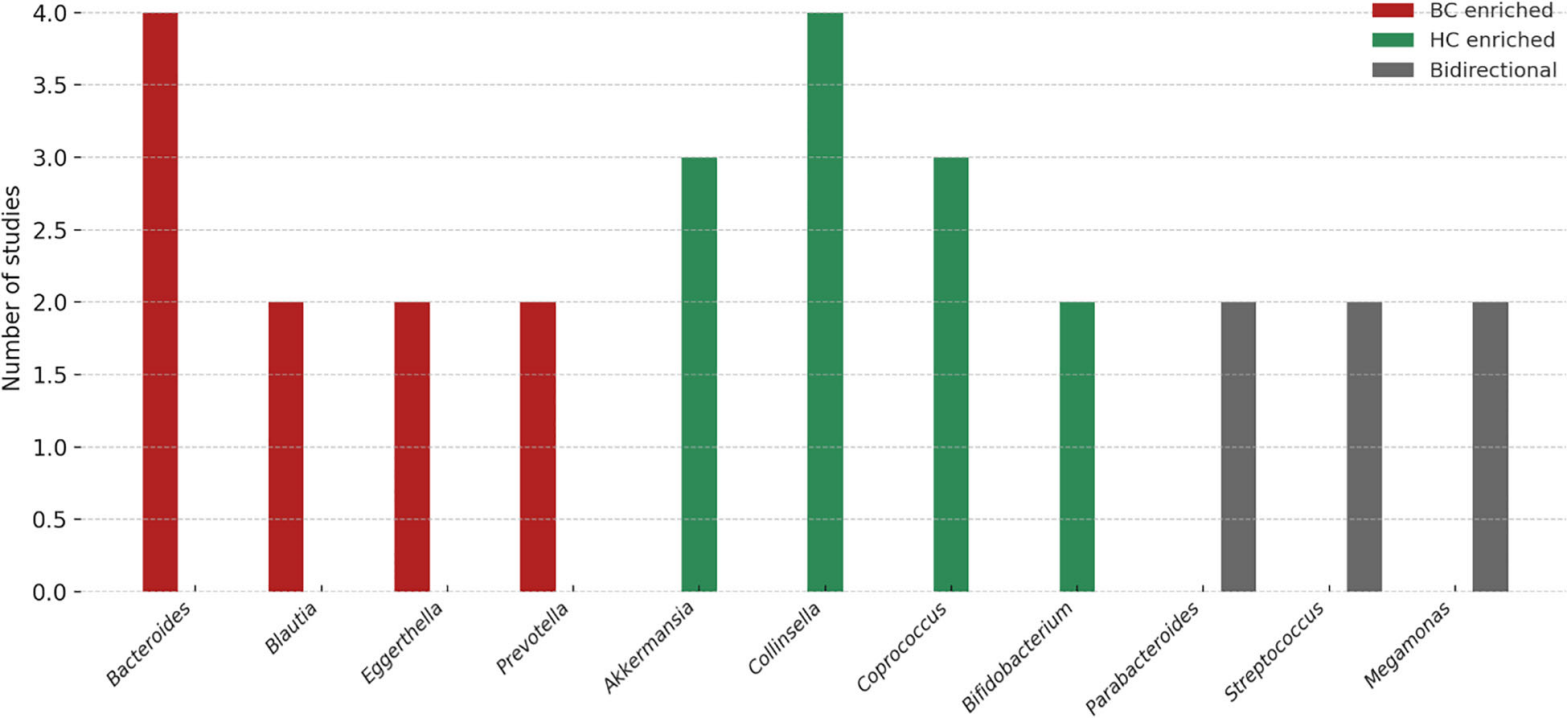
Genus-level trends in gut microbiota associated with breast cancer. The figure summarizes the number of studies reporting enrichment of specific genera in breast cancer (BC; red), healthy controls (HC; green), or with bidirectional/inconsistent associations (gray). *Bacteroides, Blautia, Eggerthella*, and *Prevotella* were more frequently reported as enriched in BC, whereas *Akkermansia, Collinsella, Coprococcus*, and *Bifidobacterium* were commonly enriched in HC. Genera such as *Parabacteroides, Streptococcus*, and *Megamonas* showed bidirectional associations, indicating possible context-dependent effects. These variations highlight both reproducible microbial signals and methodological or population-related heterogeneity that warrant validation in larger, standardized cohorts.

“At the genus level, several taxa showed consistent patterns across studies (**Figure 1**). *Bacteroides* was enriched in BC in four studies (28, 32–34), while *Collinsella* was reduced in four studies (23, 24, 33, 34), suggesting potential roles as risk- and protective-associated genera, respectively. *Blautia* (31, 32), *Eggerthella* (28, 29), *Peptoniphilus* (24, 28), *Actinomyces* (28, 33), and *Tyzzerella* (26, 27) were each reported as increased in BC in at least two independent cohorts. Conversely, *Akkermansia* (25, 33), *Coprococcus* (28, 31, 34), and occasionally *Collinsella* (24, 32, 33) were more abundant in healthy controls, suggesting protective associations. Several genera displayed bidirectional findings across populations: *Megamonas* (2↑, 2↓) (23, 24, 28, 33), *Parabacteroides* (2↑, 1↓) (28, 32, 33), and *Streptococcus* (2↑, 1↓) (25, 28, 33), indicating potential context-specific influences such as diet, menopausal status, or methodology (32).

#### 3.3.3 Species level

“At the species level, some consistent patterns emerged. *Faecalibacterium prausnitzii* was consistently depleted in BC patients in multiple studies (29, 33), supporting its proposed anti-inflammatory and protective role. By contrast, *Eggerthella lenta* (29, 33) and *Parabacteroides distasonis* (32, 33) showed bidirectional associations, with some studies reporting enrichment in BC and others showing depletion. Other species, such as *Akkermansia muciniphila* (25, 33) were more frequently reported as enriched in controls, suggesting a potentially protective influence.

### 3.4 Geographical comparison of microbiota differences in BC Across China, USA, and Europe

**Figure 2:**
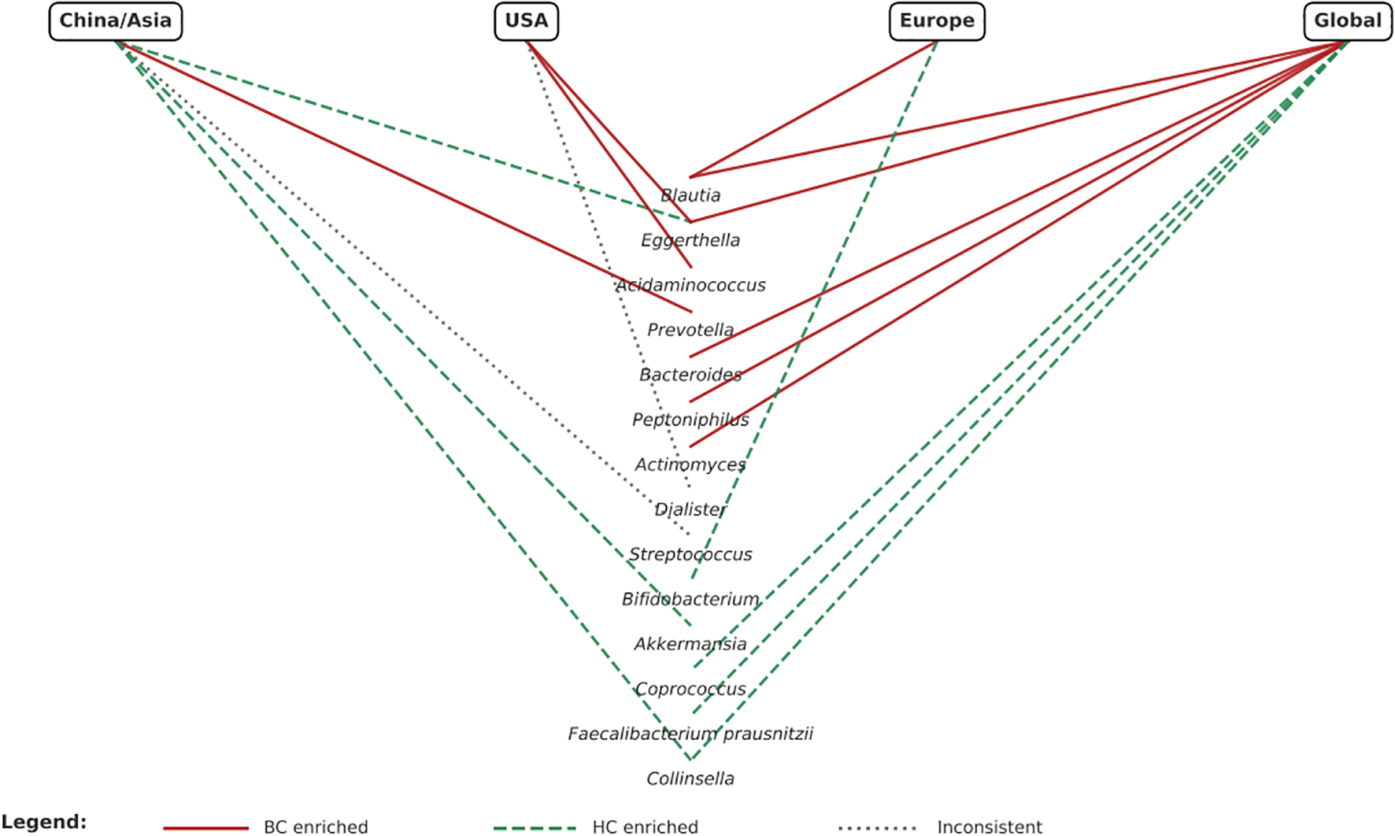
Geographical variations.

Distinct geographical patterns in microbiota composition were observed (**Figure 2**). In Chinese cohorts, *Prevotella* was enriched in BC patients (24, 33), while *Akkermansia* (25, 33) and *Collinsella* (23, 24, 33) were generally depleted, suggesting possible protective roles. However, findings for Streptococcus were inconsistent, with one study showing an increase (25) and another a decrease (33). In the USA, *Acidaminococcus* (27, 28)and *Eggerthella* (28, 29) were consistently enriched in BC patients, whereas *Dialister* showed contradictory patterns (27, 28). European studies reported enrichment of *Blautia* (31, 32) in BC and depletion *of Bifidobacterium* (31, 32) compared with controls. When considered together, Asian cohorts tended to show enrichment of *Prevotella* and depletion of *Akkermansia/Collinsella*, whereas Western cohorts more often reported enrichment of *Blautia*, *Acidaminococcus*, and *Eggerthella*. These differences likely reflect not only biological variation but also dietary patterns (e.g., high-fiber traditional Asian diets *vs* higher fat Western diets), ethnicity-related host–microbiome interactions, antibiotic use, and methodological heterogeneity. These confounders must be critically accounted for before geographical differences can be translated into predictive or therapeutic applications.

## 4 Discussion

This review synthesizes emerging evidence on gut microbiota alterations in BC, with a particular emphasis on geographical variation across China, the USA, and Europe. To our knowledge, this is the first review to explicitly examine regional differences in gut microbiome cancer associations while incorporating species-level insights. Our findings highlight consistent signs of dysbiosis, identify potential candidate biomarkers, and point to opportunities for translational application, consistent with previous studies (35–37). At the same time, they underscore the methodological heterogeneity, modest evidence base, and predominance of cross-sectional designs that constrain definitive conclusions (19, 23, 34).

Overall, alpha diversity was reduced in BC patients in seven of the thirteen included studies (24, 25, 27, 28, 31, 33, 34), suggesting disrupted microbial homeostasis and a possible link to systemic inflammation (38, 39). However, two studies found increased alpha diversity (19, 26) and four reported no significant differences (23, 29, 30, 32). Beta diversity findings were similarly inconsistent; seven studies reported significant community-level shifts (19, 23–25, 28, 29, 33), whereas others observed no significant differences. These conflicting results likely reflect heterogeneity in study populations, particularly menopausal status, as well as methodological variability in sequencing approaches and the use of different diversity indices (40–42). Standardization of analytic pipelines will be essential to allow comparability across studies.

At the phylum level, four studies observed increased F/B ratios in BC patients (23, 26, 29, 31), consistent with pro-inflammatory states and altered energy harvesting (41, 43). In contrast, three studies reported no difference or reduced ratios (24, 25, 28), highlighting the influences of dietary and methodological influences (44, 45). These discrepancies highlight the limited utility of broad phylum-level metrics as reliable biomarkers. Such conflicting findings may reflect heterogeneity in host factors and study design, including dietary patterns (e.g., high-fiber *vs*. Western diets), sequencing approaches (16S rRNA *vs*. shotgun metagenomics), and menopausal status, which shapes the hormonal and metabolic milieu. Notably, compositional variability at the taxonomic level may converge functionally, through shared microbial outputs such as short-chain fatty acids or estrogen-modulating enzymes (10, 46). This functional redundancy suggests that integrative approaches combining compositional and metabolomic analyses are essential to elucidate the biological relevance of microbiome alterations in BC. More informative trends emerged at the genus level. Enrichment of *Bacteroides*, *Blautia*, *Eggerthella* and *Parabacteroides* in BC patients was observed across multiple studies (23, 24, 28, 29, 31, 34). These genera are linked to bile acid metabolism, pro-inflammatory signaling, and estrogen reactivation (47, 48). *Eggerthella*, in particular, is notable for β-glucuronidase activity, which may increase circulating bioactive estrogens and drive tumor progression (38, 49). Conversely, protective taxa, including *Akkermansia*, *Collinsella*, and *Coprococcus* were consistently depleted (19, 24, 25, 32–34). Of particular note, *Akkermansia muciniphila* was frequently reduced, consistent with its established role as a marker of mucosal health (50).

Species-level analysis, although limited to three metagenomic studies (23, 31, 32), yielded greater biomarker specificity. *Faecalibacterium prausnitzii*, a key butyrate producer with anti-inflammatory properties, was consistently reduced in BC patients (29, 33). By contrast, *Eggerthella lenta* and *Parabacteroides distasonis* showed inconsistent patterns of enrichment and depletion across cohorts (29, 31–33). These bidirectional results may reflect population-level dietary differences, strain-level functional variation, or technical inconsistencies (51, 52). Importantly, different taxonomic changes may converge on similar functional outcomes, such as reduced SCFA production or enhanced estrogen reactivation, suggesting that functional signatures may prove more reliable than taxonomy alone (53–55).

A major and novel contribution of this review is the comparative analysis of geographical variation. In the United States, enrichment of *Acidaminococcus* and *Eggerthella* was consistently reported (27–29), plausibly linked to high-fat, high-protein dietary patterns (45, 56). In Europe, *Blautia* enrichment was observed (31, 32), suggesting that fiber-driven microbial fermentation may influence breast (57, 58). In Chinese cohorts, enrichment of *Prevotella* and depletion of *Akkermansia* and *Collinsella* were common (19, 24, 25, 32, 33, 59, 60) reflecting carbohydrate-rich and fermented food dietary profiles (44, 45). Taken together, Asian cohorts showed *Prevotella* dominance and reduced SCFA-producing taxa, whereas Western cohorts were more likely to report enrichment of *Blautia, Acidaminococcus, and Eggerthella*. These patterns highlight how diet, ethnicity, and host factors interact with microbial ecology. However, translation into clinical application is premature. Regional differences complicate biomarker standardization but also create opportunities for precision nutrition and region-specific interventions (9, 61, 62). Contradictory findings, such as divergent F/B ratios or variable *Collinsella* levels, require careful appraisal. These discrepancies can be explained by methodological heterogeneity, including differences in DNA extraction, sequencing platform, and analytic pipelines. Clinical and demographic confounders, such as menopausal status, body mass index, antibiotic exposure, and treatment history, were often incompletely reported yet are known to shape microbiome composition. Importantly, taxonomic variability may still converge on functional similarity: reduced SCFA production, loss of barrier integrity, and enhanced estrogen metabolism are recurrent themes. Future work must therefore integrate metagenomics, metabolomics, and metatranscriptomics to link compositional changes with mechanistic outputs (8).

The potential of the gut microbiome as a predictive biomarker in BC should be viewed as preliminary. Current evidence is largely cross-sectional and insufficient to establish causality. Nevertheless, recurring signals, such as depletion of *Faecalibacterium prausnitzii* and *Akkermansia muciniphila*, provide biologically plausible candidate biomarkers that merit validation (42, 50, 63). Integration with established predictors, including circulating estrogen, inflammatory markers, hormone receptor status, and genomic risk scores, could yield multi-modal models with greater predictive power (64, 65). In practice, stool-based microbiome profiling could emerge as a low-cost, non-invasive adjunct, but clinical application will require reproducible assays, validated thresholds, and demonstration of incremental benefits (9, 49). Microbiota-based interventions represent a promising but as yet untested avenue in BC (63, 66). Evidence from other cancers indicates that dietary fiber, probiotics, and fecal microbiota transplantation can modulate therapeutic response (62, 66, 67). In breast cancer, dietary modification, particularly fiber enrichment or polyphenol supplementation, may support protective taxa such as *Faecalibacterium and Akkermansia*. Probiotic and prebiotic interventions targeting estrogen metabolism or SCFA production are theoretically attractive but require robust testing in controlled trials (50, 63, 66).

This review has several strengths, including its structured literature search, inclusion of species-level analyses, and integration of geographical perspectives, which have been largely overlooked in prior work. However, important limitations must be acknowledged. Only 13 studies met eligibility criteria, reflecting the early stage of this field. Most were small, cross-sectional studies relying on 16S rRNA sequencing, which restricts taxonomic resolution. Only three employed metagenomic approaches, which are needed for functional insight. Menopausal status, body mass index, and antibiotic use were inconsistently reported, limiting comparability. Restriction to English-language publications may have introduced selection bias. A formal risk-of-bias assessment was not conducted, consistent with the Mini Review format, but methodological variability was qualitatively addressed.

Future research should therefore prioritize prospective designs to establish temporal and causal relationships between dysbiosis and breast cancer (68). Microbiome signatures should be integrated with established biomarkers in multivariable models to test whether they improve prediction. Region-specific interventions should be trialed, recognizing that microbiome–diet interactions are culturally and geographically contingent (13). Functional profiling must be incorporated to reconcile taxonomic heterogeneity and clarify biological plausibility. Harmonization of methods for sampling, sequencing, and analysis will be critical to reproducibility (69). Finally, international collaborations are needed to validate microbial predictors across diverse populations, including underrepresented regions such as Africa and South America, ensuring equitable global translation (61, 67, 70).

In conclusion, while the gut microbiome cannot yet be regarded as an established predictive biomarker for BC, the trajectory of current research suggests considerable promise. Consistent signals at genus and species levels, functional links to estrogen metabolism and inflammation, and region-specific variation provide a biologically credible foundation for further study. Translation into clinical practice will depend on large-scale, longitudinal, standardized studies capable of establishing causality and reproducibility. If achieved, microbiome-informed approaches may ultimately contribute to precision oncology by enhancing risk stratification, guiding dietary counselling, and supporting the development of microbiome-targeted interventions.
